# Monoterpenoid Epoxidiol Ameliorates the Pathological Phenotypes of the Rotenone-Induced Parkinson’s Disease Model by Alleviating Mitochondrial Dysfunction

**DOI:** 10.3390/ijms24065842

**Published:** 2023-03-19

**Authors:** Yulia Aleksandrova, Kirill Chaprov, Alexandra Podturkina, Oleg Ardashov, Ekaterina Yandulova, Konstantin Volcho, Nariman Salakhutdinov, Margarita Neganova

**Affiliations:** 1 Institute of Physiologically Active Compounds at Federal Research Center of Problems of Chemical Physics and Medicinal Chemistry, Russian Academy of Sciences, Severnij Pr. 1, Chernogolovka 142432, Russia; 2 Department of Medicinal Chemistry, N. N. Vorozhtsov Novosibirsk Institute of Organic Chemistry, Siberian Branch, Russian Academy of Sciences, Lavrentiev Ave., 9, Novosibirsk 630090, Russia

**Keywords:** epoxidiol, Parkinson’s disease, neurodegenerative diseases, rotenone, ROS, mitochondrial dysfunction, disease-modifying therapy

## Abstract

Parkinson’s disease is the second most common neurodegenerative disease. Unfortunately, there is still no definitive disease-modifying therapy. In our work, the antiparkinsonian potential of trans-epoxide (1S,2S,3R,4S,6R)-1-methyl-4-(prop-1-en-2-yl)-7-oxabicyclo [4.1.0]heptan-2,3-diol (E-diol) was analyzed in a rotenone-induced neurotoxicity model using in vitro, in vivo and ex vivo approaches. It was conducted as part of the study of the mitoprotective properties of the compound. E-diol has been shown to have cytoprotective properties in the SH-SY5Y cell line exposed to rotenone, which is associated with its ability to prevent the loss of mitochondrial membrane potential and restore the oxygen consumption rate after inhibition of the complex I function. Under the conditions of rotenone modeling of Parkinson’s disease in vivo, treatment with E-diol led to the leveling of both motor and non-motor disorders. The post-mortem analysis of brain samples from these animals demonstrated the ability of E-diol to prevent the loss of dopaminergic neurons. Moreover, that substance restored functioning of the mitochondrial respiratory chain complexes and significantly reduced the production of reactive oxygen species, preventing oxidative damage. Thus, E-diol can be considered as a new potential agent for the treatment of Parkinson’s disease.

## 1. Introduction

Parkinson’s disease is the second most common disease among neurodegenerative disorders [1,2] after Alzheimer’s disease [3] and is characterized primarily by motor disorders caused by the loss of dopaminergic neurons in the compact part of the substantia nigra [4,5]. In patients with advanced stages of this disease, up to 95% of these neurons die [6]. A large number of papers describing the possible mechanisms of dopaminergic neurons loss are devoted to the study of the mitochondrial complex I (NADH-dehydrogenase complex) activity, a decrease in the activity of which is shown in the brain of patients with parkinsonism [7,8,9]. This complex is the main entry point of electrons into the respiratory chain, by which initiates oxidative phosphorylation and the ATP production by mitochondria [10]. Given that an impairment in the functioning of mitochondria currently plays a key role in the pathogenesis of Parkinson’s disease [11,12], a promising direction in the search for potential drugs is to focus on their ability to level mitochondrial dysfunction.

**Figure 1:**
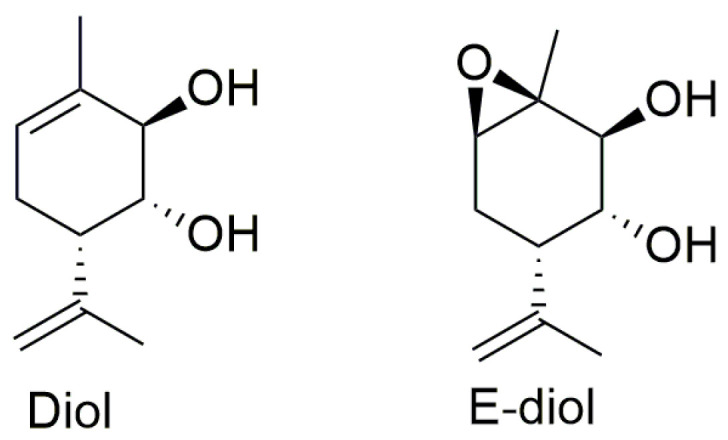
Structures of Diol and E-diol.

A promising class of compounds on the basis of which highly effective neuroprotective drugs for the treatment of Parkinson’s disease can be developed are monoterpenoids [13,14,15,16]. Previously, the antiparkinsonian potential of (1R,2R,6S)-3-methyl-6-(prop-1-en-2-yl)cyclohex-3-en-1,2-diol (diol (Prottremin), Figure 1) [17,18], which is currently in the first stage of clinical trials, was discovered. The active metabolite epoxide (1S,2S,3R,4S,6R)-1-methyl-4-(prop-1-en-2-yl)-7-oxabicyclo[4.1.0]heptane-2,3-diol (E-diol, shown in Figure 1) was also found to produce a similar effect as the compound mentioned earlier. E-diol has a high antiparkinsonian activity. In addition, epoxidiol has demonstrated the ability to repair dopaminergic neurons damaged by the neurotoxin MPTP, triggering a signaling cascade of mitogen-activated protein kinase (MAPK) [19]. It gives hope for an effective treatment of the disease. In order to bring more insight into possible mechanisms of the antiparkinsonian action of epoxidiol, in this work, we conducted for the first time a study of its biological activity as part of a consecutive study of mitoprotective properties at in vitro, in vivo and ex vivo testing stages.

**Figure 2:**
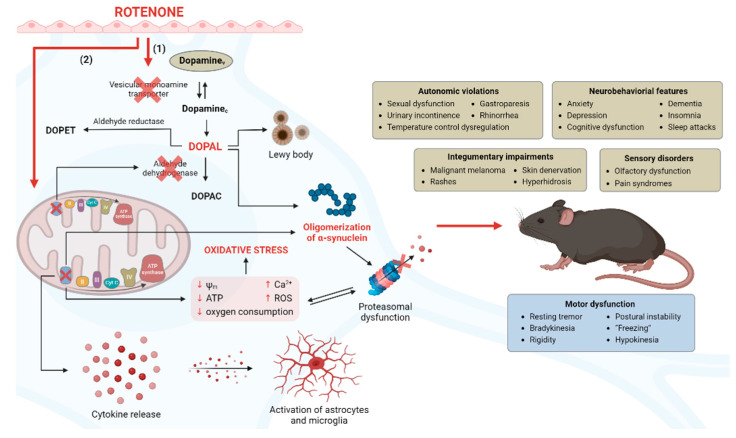
The proposed mechanism of rotenone action in the parkinsonism progression. Within the neuron, dopamine exists in two forms: vesicular (Dopamine_v_) and cytoplasmic (Dopamine_c_), the balance of which is maintained due to the functioning of the vesicular monoamine transporter (VMAT). VMAT promotes the active deposition of the neurotransmitter in vesicles. Dopamine_c_ in neurons undergoes oxidative deamination, catalyzed by monoamine oxidase A, to form DOPAL. DOPAL is a cytotoxic catecholaldehyde and promotes the formation of alpha-synuclein oligomeric forms, pathogenic in Parkinson’s disease. However, under normal conditions, DOPAL metabolism is regulated by enzymes, mainly aldehyde dehydrogenase, resulting in the formation of a non-toxic metabolite DOPAC, which helps to reduce the level of inflammatory cytokines. The rotenone introduction leads to an increase in the content of endogenous DOPAL, which correlates with the increased death of neuronal cells and neurobehavioral abnormalities similar to those in Parkinson’s disease. This can happen for two reasons. (1) Rotenone, acting on VMAT, blocks it, and as a result, dopamine redistribution occurs only in one direction—from vesicles to the cytoplasm. Such a shift of Dopamine_c_ > Dopamine_v_ leads to the increased availability of dopamine as a precursor for the production of DOPAL in abnormal amounts. (2) Rotenone, by blocking the mitochondrial complex and, as a consequence, the generation of NAD^+^, can reduce the intracellular activity of aldehyde dehydrogenase, since NAD^+^ is a necessary cofactor for the functioning of this enzyme. This leads to an increase in the DOPAL level, as now its metabolism is regulated using only an alternative pathway through aldoreductase.

To simulate the pathogenesis of Parkinson’s disease, rotenone was used, which is a widely used neurotoxin in a large number of studies. It is used both to elucidate the mechanisms underlying the death of dopaminergic cells and to study new potential neuroprotective agents [20,21,22,23]. This is due to its involvement in many pathological pathways that mediate the death of dopaminergic neurons [24,25,26], the ability to reproduce both motor [27,28] and non-motor symptoms of parkinsonism [29,30,31], as well as an extremely high lipophilicity [32], which allows it to penetrate easily the blood–brain barrier. Figure 2 shows the mechanisms of rotenone action that cause neuropathological signs, as well as motor and non-motor symptoms. It has been proven that rotenone reproduces the most common symptoms of Parkinson’s disease due to direct inhibition of the mitochondrial respiratory chain complex I [33,34].

In our work, we tried to reproduce a rotenone-induced model of Parkinson’s disease using in vitro, in vivo and ex vivo approaches. It was investigated whether treatment with epoxidol by modulating mitochondrial functions could restore the behavioral and neurochemical profile of mice with the phenotype of this disease.

## 2. Results

### 2.1. Rotenone-Induced Neurotoxicity on the SH-SY5Y Cell Line

**Figure 3:**
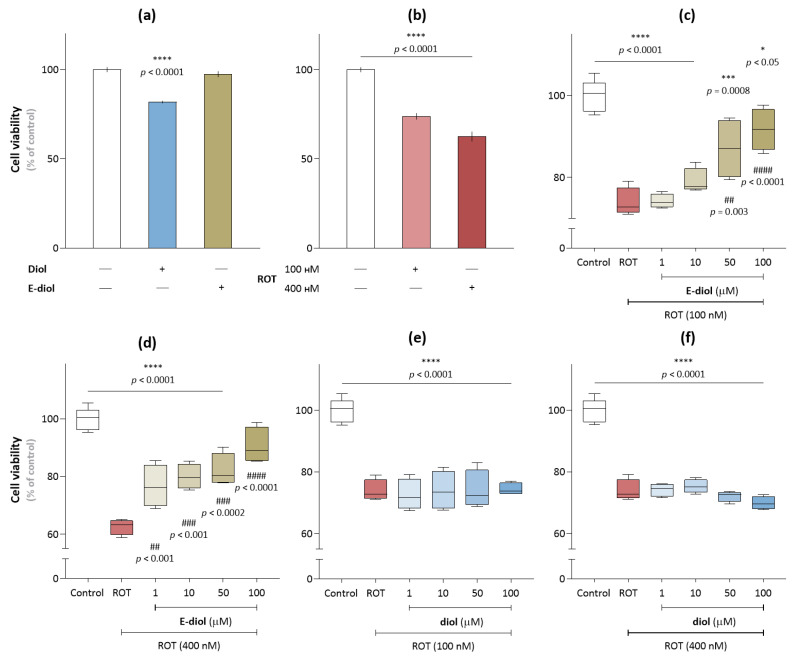
Viability of SH-SY5Y cells assessed by MTT analysis. Analysis of the viability of SH-SY5Y cells (10,000 cells per well) in the presence of (**a**) the studied compounds and (**b**) rotenone. The effect of diol and epoxidiol in the concentration range from 1 µM to 100 µM on the survival of SH-SY5Y cells in the rotenone presence at concentrations of 100 nM (**c**, **e**) and 400 nM (**d**, **f**). The data are presented as an average ± SEM. ****, *p* ≤ 0.0001, ***, *p* ≤ 0.001 and *, *p* ≤ 0.05 versus control; ^####^, *p* ≤ 0.0001, ^###^, *p* ≤ 0.001 and ^##^, *p* ≤ 0.01 (one-way ANOVA and Dunnett’s multiple comparison tests).

To assess the effect of diol (initial compound) and epoxidiol on the viability of SH-SY5Y cells, an MTT analysis was performed based on the ability of mitochondrial dehydrogenases of living metabolically active cells to cleave the membrane-permeable yellow tetrazolium salt (3-[4,5-dimethylthiazole-2-yl]-2,5-diphenyltetrazolium bromide, MTT), restoring it to purple intracellular formazan crystals. Initially, to assess the possible intrinsic toxic effects of the compounds under study, their effect on cell survival was tested at the maximum used concentration of 100 µM in absence of rotenone. It was found that diol leads to suppression of cell survival by 18.13 ± 0.63% (*p* < 0.0001), but its epoxide did not cause any decrease in cell viability (Figure 3a) (97.48 ± 0.93, *p* = 0.43). In turn, the selected concentrations of rotenone (100 nM and 400 nM) led to a decrease in cell survival by 26.13 ± 1.75% and 37.40 ± 1.34% (*p* < 0.0001) (Figure 3b) compared with control samples, which is consistent with the already known data obtained for this toxin in similar experiments [35,36].

In experiments with the combined use of the studied compounds and rotenone, it was found that epoxidiol showed a protective effect on the SH-SY5Y cell line exposed to rotenone (Figure 3c,d). This effect was concentration-dependent, reaching a maximum at 100 µM and increasing the number of viable cells from 73.87 ± 1.75% (rotenone concentration—100 nM) and 62.60 ± 1.34% (rotenone concentration—400 nM) up to 91.74 ± 2.54% and 90.53 ± 3.18% (*p* < 0.0001), respectively. At the same time, diol had no effect on the viability of cells treated with rotenone (Figure 3e,f).

### 2.2. Rotenone-Mediated Depolarization of Isolated Rat Liver Mitochondria

**Figure 4:**
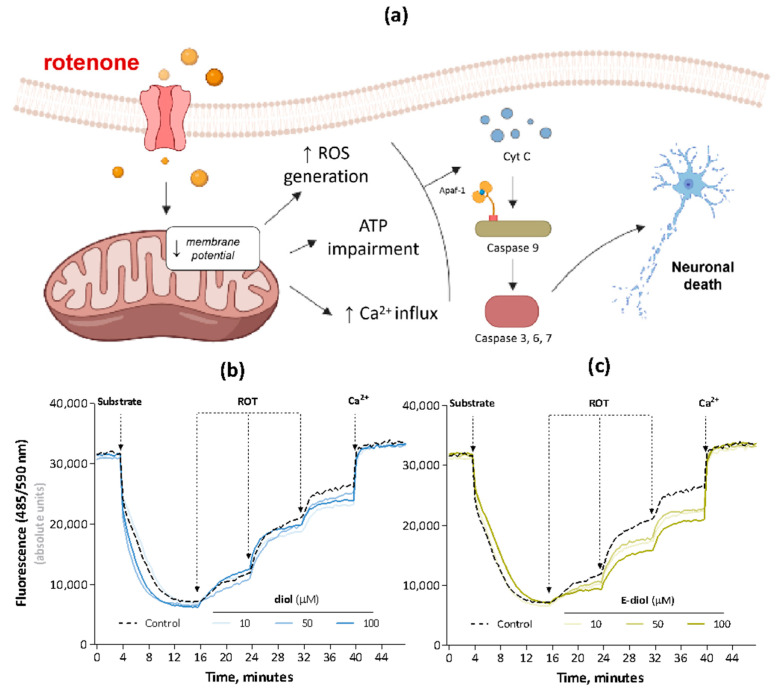
Schematic representation of the rotenone effect on the mitochondrial transmembrane potential, leading to the death of neuronal cells (**a**). Evaluation of the mitochondrial membrane potential using the fluorescent lipophilic cation safranin O. The effect of diol (**b**) and epoxidiol (**c**) in the concentration range from 10 µM to 100 µM on the transmembrane potential of rat liver mitochondria (0.5 mg/mL) in the rotenone presence at a concentration of 10 nM. Energization of organelles was carried out by substrates of the electron transport chain I complex—glutamate/malate (5 mM). The achievement of complete depolarization was stimulated by Ca^2+^ ions (25 µM). The data are presented as kinetic curves of changes in the mitochondrial membrane potential (mean ± SEM).

To analyze the process of depolarization of the mitochondrial membrane under the action of rotenone, the transmembrane potential of organelles pretreated with the studied compounds in the concentration range from 10 to 100 µM was measured. A potential-dependent safranin O label was used, the fluorescence of which is quenched in the mitochondrial matrix of polarized organelles [37]. The value of the transmembrane potential is inversely proportional to the values of the safranin O fluorescence. The kinetic curves of changes in the mitochondrial membrane potential in samples by the action of modulators are shown in Figure 4b,c. Energization of organelles by substrates of the respiratory chain complex I—glutamate and malate led to a decrease in fluorescence, which corresponds to an increase in the transmembrane potential and reflects the use of a proton gradient to stimulate ATP synthesis. As expected, the sequential addition of rotenone led to a significant decrease in the transmembrane potential, which indicates the depolarization of the mitochondrial membrane. In turn, for the studied compounds, the ability to reduce the response of organelles to the rotenone pulses was observed. Epoxidiol most effectively normalized the impairment of the mitochondrial membrane potential caused by the toxin. This compound in the maximum studied concentration prevented the amplification of the fluorescence signal by 47% (after the first ROT injection), 37% (after the second ROT injection) and 26% (after the third ROT injection).

### 2.3. Bioenergetic Profile of the SH-SY5Y Cell Line under Conditions of Reduced Mitochondrial Function Caused by Rotenone

**Figure 5:**
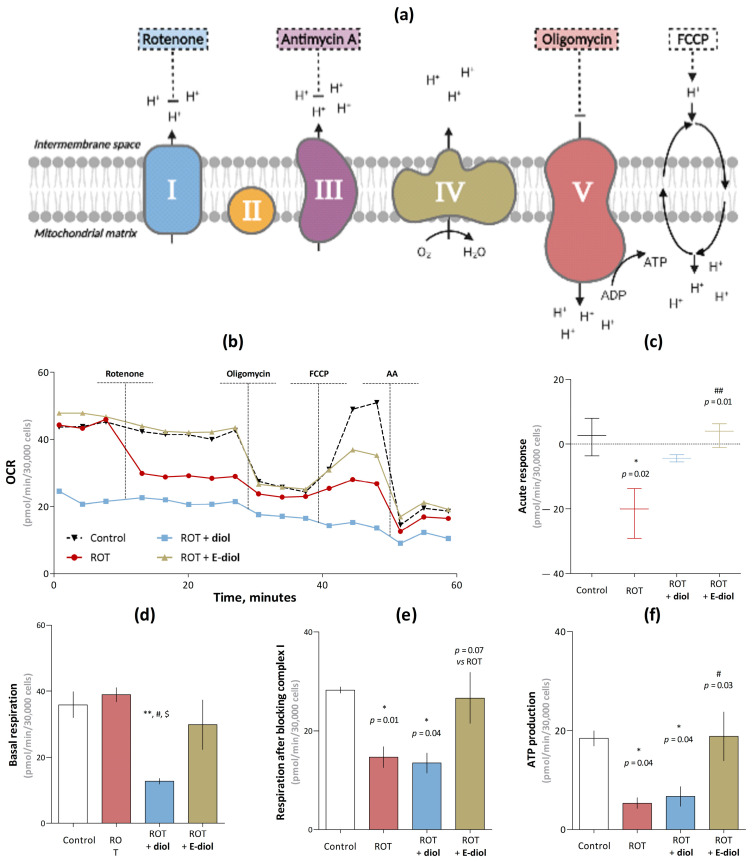
Assessment of respiratory profile and mitochondrial bioenergetic characteristics of neuroblastoma cell culture SH-SY5Y using Seahorse XF96 Extracellular Flux Analyzer. Schematic representation of the mitochondrial respiratory chain complexes work and the impact of the Seahorse/Agilent Mito Stress Test modulators (**a**). Kinetic curves of changes in the oxygen consumption rate of the SH-SY5Y neuroblastoma cell culture (30,000 cells per well) pretreated within 24 h by the studied compounds over time under the modulators action (**b**). The concentrations of diol and epoxidiol were 100 µM, rotenone—10 nM, oligomycin—2 µM, FCCP—2 µM and antimycin A—1 µM. Data are shown as mean ± SEM. Bioenergetic parameters of mitochondria, such as acute response to the first injection of rotenone (**c**); basal respiration, which is the oxygen consumption rate before the introduction of modulators into the system (**d**); respiration after blocking the NADH-dehydrogenase complex of the electron transfer chain by rotenone (**e**); as well as ATP production, which is the difference between the last OCR measurement before the oligomycin injection and the minimum OCR after the oligomycin injection (**f**). The data are presented as graphs in which each column represents the average value of an independent cell population (mean ± SEM, *n* = 6). To assess the statistical significance, one-way ANOVA and Dunnett’s multiple comparison tests were used, where * *p* ≤ 0.05 and ** *p* ≤ 0.01 versus control; # *p* ≤ 0.05 and ## *p* ≤ 0.01 versus ROT; $ *p* ≤ 0.05 versus ROT + E-diol.

To study the bioenergetics of mitochondria, a Seahorse/Agilent Mito Stress Test was used on Seahorse XFe96 Extracellular Flux Analyzer [38] with some modifications. Before starting the analysis according to the standard protocol, neuroblastoma cells were subjected to 24 h treatment with the test compounds at a 100 µM concentration. Interestingly, the oxygen consumption rate (OCR) in the analysis of basal respiration was the same for all groups with the exception of diol, which significantly reduced it (Figure 5b). After the first injection, a significant decrease in the oxygen consumption rate from 28.22 ± 0.65 pmol/min to 14.68 ± 2.11 pmol/min was observed in cells treated with a non-toxic 10 nM rotenone concentration compared to control cells receiving solvent injection (on average by 48%, *p* = 0.01, Figure 5b,e). It was also confirmed by analyzing the quantitative indicator of the acute response parameter (Figure 5c). However, pretreatment of neuroblastoma cells with epoxidol was able to neutralize the effects caused by the toxin (Figure 5b,e). The OCR of the ROT + E-diol group was at the level of control samples (26.63 ± 5.11 pmol/min) and had a strong tendency to increase compared to the ROT group (*p* = 0.07). A similar situation was observed in the OCR indicator associated with ATP production (Figure 5f). Rotenone significantly reduced this parameter from 18.43 ± 1.53 pmol/min to 5.33 ± 1.11 pmol/min (by 71% when compared with the control group, *p* = 0.04). Finally, the use of epoxidiol kept the OCR associated with ATP production at the level of 18.87 ± 4.93 pmol/min, which is significantly higher than in the samples with rotenone (by 72% when compared with ROT, *p* = 0.04). In the case of samples pretreated with diol at a 100 µM concentration, the OCR was already at a much lower level from the first measurement, and the subsequent addition of modulators to the medium did not cause any pronounced responses. This is most likely due to some cytotoxic activity detected above for this substance, where 18.13 ± 0.63% of cells died at this concentration (Figure 3a).

### 2.4. In Vivo Study of Motor Activity and Endurance of Mice Simulating Parkinson’s Disease

**Figure 6:**
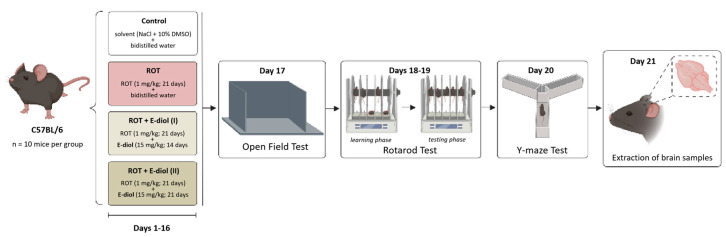
An in vivo scheme of the series of experiments aimed at investigating the neuroprotective potential of epoxidiol (E-diol, 15 mg/kg) with intraperitoneal administration (i. p.) under conditions of rotenone-induced model (ROT, 1 mg/kg, i. p.) neurotoxicity.

To simulate the pathological phenotype of Parkinson’s disease with the in vivo studies, male mice of the C57BL/6J line were injected with rotenone at a 1 mg/kg dose daily for 21 days by intraperitoneal injection (Figure 6). In order to compare possible differences in the neuroprotective effects of epoxidiol, the compound at a 15 mg/kg dose was administered according to two schemes: (1) daily, starting from the 8th day of the experiment in already formed pathology conditions, and (2) daily throughout the entire period of the experiment. As a control group, animals of the same age were used, which received injections of equivalent volumes of solvents.

**Figure 7:**
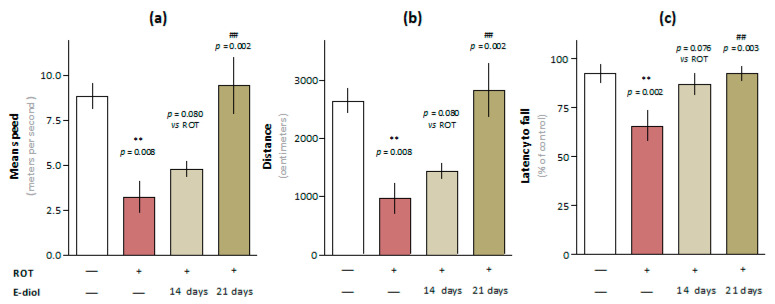
In vivo study of the motor function of C57BL/6J mice aged 3 months (*n* = 10) in the Open Field test: average speed (**a**) and distance traveled (**b**). The data are presented as graphs in which each column represents the average value of an independent mice group (mean ± SEM). Evaluation of the E-diol effect on motor coordination disorders of mice in the accelerating speed Rotarod test (**c**). The data are presented as latency to fall from rolling rod (mean ± SEM of time intervals from the beginning of the test to the fall of the animal from rolling rod for each experimental group). To assess the statistical significance, one-way ANOVA and Bonferroni’s multiple comparison tests were used, where ** *p* ≤ 0.01 versus control; ## *p* ≤ 0.01 versus ROT.

The motor characteristics of the animals were evaluated in the Open Field test by the average speed and distance traveled during a 5-min experiment. As shown in Figure 7a,b, in the ROT group there was a statistically significant decrease in the average movement speed of animals compared to the control group from 8.83 ± 0.71 m/s to 3.23 ± 0.89 m/s (*p* = 0.008 vs. Control). A similar pattern was shown in the distance traveled, which was reduced from 2645.41 ± 213.47 cm to 969.46 ± 265.69 cm (*p* = 0.008 vs. Control). Animal groups treated with epoxidiol in addition to the toxin demonstrated the ability to restore motor activity indicators. This was expressed in the tendency of mice from the ROT + E-diol (I) group to increase the average speed and distance traveled (*p* = 0.080 vs. ROT). In the ROT + E-diol (II) group, there was a significant improvement in these indicators to 9.43 ± 1.58 m/sec and 2827.26 ± 472.78, which corresponds to the level of control animals (*p* = 0.002 vs. ROT and *p* > 0.999 vs. Control).

Mice motor coordination and endurance were evaluated using an accelerating speed Rotarod test. Interestingly, most of the animals from the control group successfully passed the 5-min test during the testing phase, demonstrating almost 100% stay on the rolling rod (Figure 7d). In turn, mice simulating Parkinson’s disease spent significantly less time on rotarode than intact animals, reducing this indicator from 94.73 ± 3.52% to 68.87 ± 7.12% (*p* = 0.002 vs. Control). This indicates that the mice motor function from the ROT group was significantly impaired, which was expressed in their inability to stay on the rolling rod. Treatment with epoxidiol significantly improved the ability of animals to stay on the rotarode, while, as in the Open Field test, when using the first administration scheme, there was a tendency to improve endurance and coordination (86.13 ± 3.98%, *p* = 0.076 vs. ROT). Combination therapy with epoxidiol during the entire period of the in vivo experiment led to a significant increase in the time spent on rotarode—94.03 ± 2.78% (*p* = 0.002 vs. ROT) up to the level of control mice (*p* > 0.999 vs. Control).

### 2.5. In Vivo Study of Hippocampus-Dependent Spatial Memory of Mice Simulating Parkinson’s Disease

**Figure 8:**
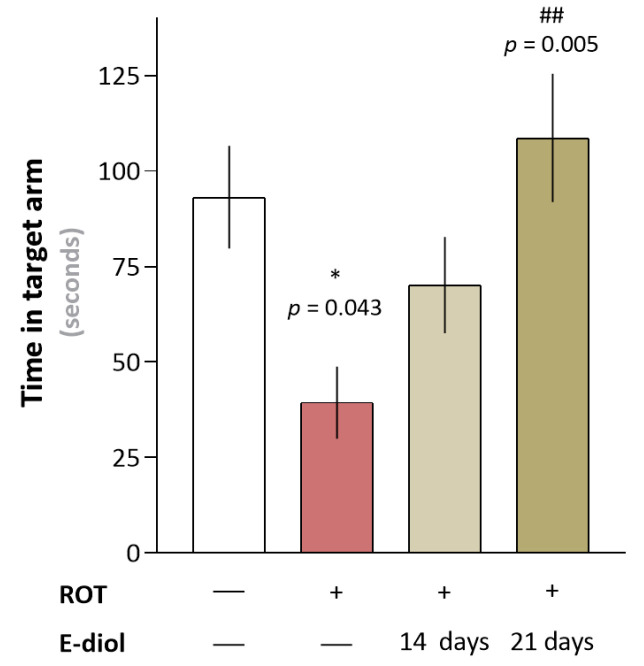
In vivo study of the epoxidiol (E-diol) effect on the formation of short-term spatial memory in C57BL/6J mice aged 3 months under conditions of rotenone-induced (ROT) neurotoxicity. The data are presented as graphs in which each column represents the average time spent by an independent mice group in the correct arm of the maze (mean ± SEM). To assess the statistical significance, one-way ANOVA and Bonferroni’s multiple comparison tests were used, where * *p* ≤ 0.05 versus control; ## *p* ≤ 0.01 versus ROT.

Hippocampus-dependent spatial working memory was evaluated by measuring the time mice spent in the target arm of the maze (Figure 8). It was found that mice receiving rotenone injections spent less time in the target arm of the maze during the testing phase compared to C57BL/6J control animals (39.38 ± 9.46 s for the ROT group and 70.13 ± 12.60 s, *p* = 0.043 vs. Control). In turn, for the ROT + E-diol (II) group, which received epoxidiol starting from the first day of the in vivo experiment, the ability to significantly increase this indicator up to 108.63 ± 16.80 s (*p* = 0.005 vs. ROT) was observed, exceeding that of the control group.

### 2.6. The Level of Dopaminergic Neurons in Brain Samples of Mice Modeling Parkinson’s Disease

To determine the number of dopaminergic neurons, brain slices were stained with an anti-tyrosine hydroxylase antibody (TH), an enzyme that limits the dopamine synthesis rate [39], and stereological counting of TH-positive neurons in substantia nigra pars compacta (SNpc) and ventral tegmental area (VTA) was performed.

**Figure 9:**
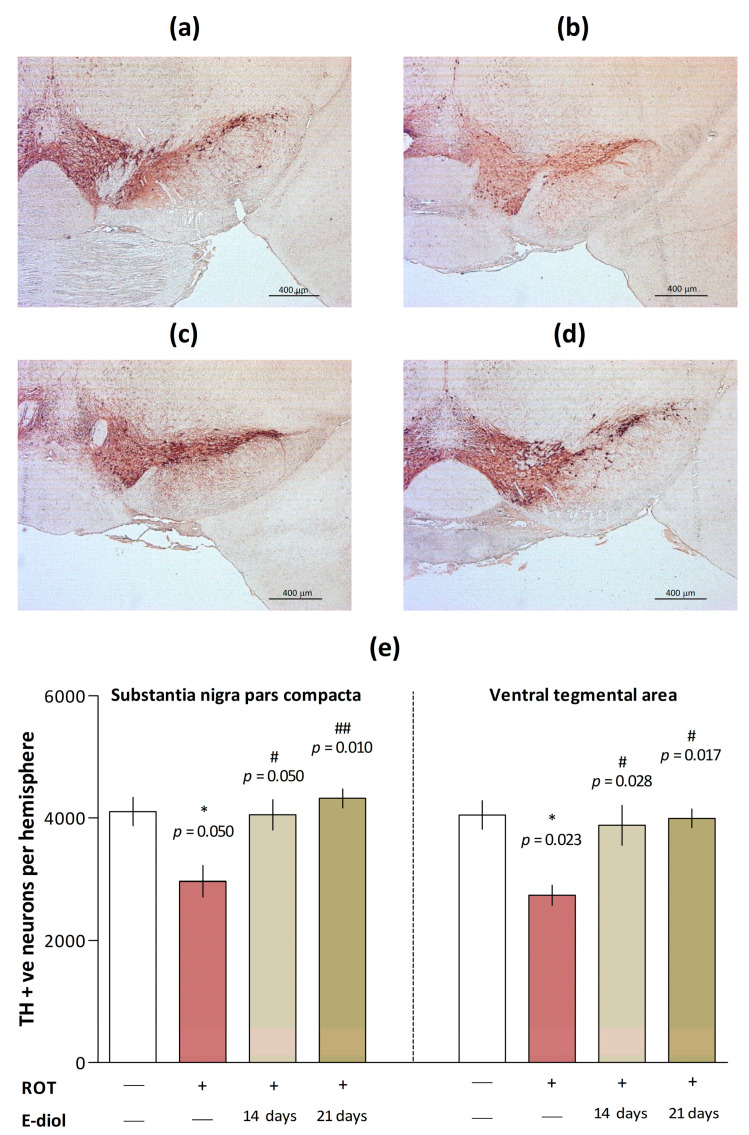
Post mortem analysis of the number of dopaminergic neurons in the mice brain regions. Representative images of brain sections of Control (**a**), ROT (**b**), ROT + E-diol (I) (**c**) and ROT + E-diol (II) (**d**) through substantia nigra pars compacta (SNpc) and ventral tegmental area (VTA) regions immunostained with an anti-tyrosine hydroxylase antibody (TH, mouse monoclonal antibody, clone TH-2, Sigma diluted 1:1000). The scale bar represents 400 μM. Histograms showing the number of TH-positive neurons in individual brain regions (**e**). The data are presented as mean ± SEM, *n* = 8. To assess the statistical significance, one-way ANOVA and Kruskal–Wallis’ multiple comparison tests were used, where * *p* ≤ 0.05 versus control; # *p* ≤ 0.05 and ## *p* ≤ 0.01 versus ROT.

In mice from the ROT group, there was a significant decrease in the number of TH-positive dopaminergic neurons in both studied areas (Figure 9b,e) by 27.8% (*p* = 0.050 vs. Control) and 32.4% (*p* = 0.023 vs. Control, Figure 9a), respectively. On the contrary, the number of neurons was significantly increased in animals treated with epoxidiol during 14 (Figure 9c,e) and 21 (Figure 9d,e) days of administration. In mice from the ROT + E-diol (I) group, this value was significantly higher by 36.6% (in SNpc; *p* = 0.050 vs. ROT) and 41.7% (in VTA; *p* = 0.028 vs. ROT). In turn, for the ROT + E-diol (II) group, the number of TH-positive dopaminergic neurons exceeded that for ROT by 45.7% (in SNpc; *p* = 0.010 vs. ROT) and 45.9% (in VTA; *p* = 0.017 vs. ROT).

### 2.7. Dynamics of Mitochondrial Respiration and Oxidative Stress in Brain Samples of Mice Modeling Parkinson’s Disease

To confirm that the symptoms of parkinsonism observed in the rotenone-induced model are due not to systemic toxicity, but to the targeted effect of the toxin on the electron transport chain, and the ability of epoxidiol to exert antiparkinsonian effects in the in vivo model of the disease is associated with the mitoprotective properties of the compound, a functional assessment of the mitochondrial complex's activity was carried out. The Seahorse XF96 cellular metabolism analyzer was used to measure the oxygen consumption rate by the mitochondrial p2 fraction obtained from the animal brain after injections of various substrates and inhibitors of electron transport chain complexes.

**Figure 10:**
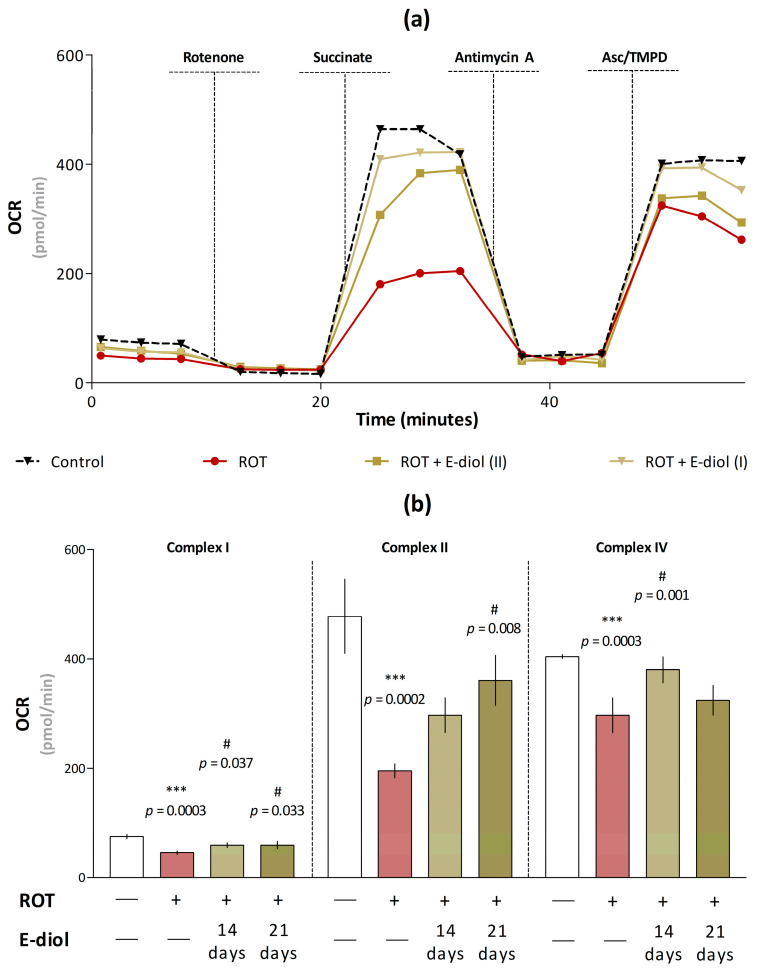
Analysis of oxygen consumption rate by mitochondrial p2 fraction using Seahorse XF96 Extracellular Flux Analyzer. Kinetic curves of changes in the oxygen consumption rate by organelles (10 micrograms per well) pretreated with glutamate and malate (10 mM) substrates of the respiratory chain I complex over time under the action of modulators (**a**). The concentration of rotenone was 2 µM, succinate—2 µM, antimycin A—1 µM and ascorbate/TMPD—0.5 µM. Data are shown as mean ± SEM. The data presented as histograms, in which each column represents the average value of the OCR by the organelles of the experimental groups (mean ± SEM, *n* = 8) (**b**). To assess the statistical significance, one-way ANOVA and Dunnett’s multiple comparison tests were used, where *** *p* ≤ 0.001 versus Control; # *p* ≤ 0.05 versus ROT.

The first three cycles of measuring the oxygen consumption rate by mitochondria pretreated with glutamate and malate substrates of complex I confirmed the hypothesis of the inhibitory effect of rotenone on NADH dehydrogenase (Figure 10a,b). This was evidenced by a decrease in this indicator from 74.78 ± 2.34 pmol/min (for control samples) to 45.73 ± 1.95 pmol/min (*p* = 0.0003 vs. Control). This effect was reduced in brain samples of animals from groups treated with epoxidiol, where exposure to the compound led to an increase in the oxygen consumption rate by 28.7% (in the case of ROT + E-diol (I); *p* = 0.037 vs. ROT) and 29.4% (for ROT + E-diol (II); *p* = 0.033 vs. ROT). After inhibition of the NADH-dehydrogenase complex by rotenone, its substrate, succinate, was added to stimulate complex II respiration. Enhanced respiration was shown for all groups; however, in the mitochondrial p2 fraction obtained in ROT mice, the activity of this complex was reduced by more than 50% (*p* = 0.0002 vs. Control). In turn, mice treated with epoxidiol for 21 days had an increase in OCR from 195.04 ± 7.336 pmol/min to 360.49 ± 26.46 (*p* = 0.008 vs. ROT). As expected, the subsequent addition of an inhibitor of complex III, antimycin A, reduced OCR, which was eliminated by the introduction of electron donors of complex IV—ascorbate/N,N,N, N-tetramethyl-p-phenylenediamine (TMPD), delivering them directly to cytochrome C oxidase. A similar situation was found in the ROT samples with a decrease in the oxygen consumption rate observed in the case of complex II. It indicates that the blocking of complex I by rotenone entails a further cascade of events that prevents the transport of electrons throughout the subsequent chain. And in this case, epoxidiol had the ability to improve mitochondrial function.

**Figure 11:**
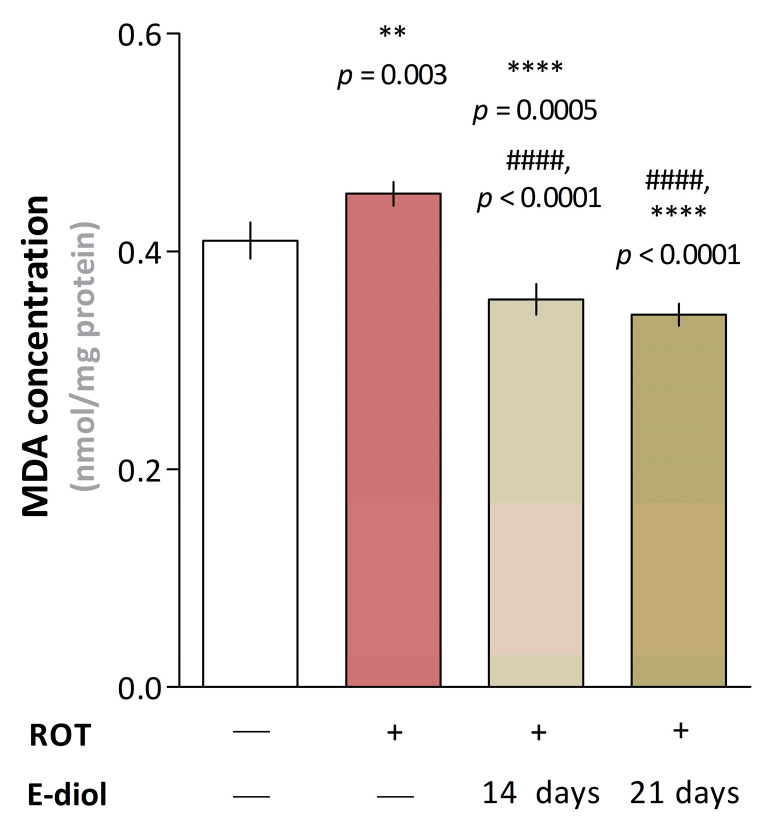
Measurement of the malondialdehyde (MDA) concentration in mouse brain homogenates (4 mg/mL). The data are presented as histograms showing the malondialdehyde amount as mean ± SEM, *n* = 8. To assess the statistical significance, one-way ANOVA and Dunnett’s multiple comparison tests were used, where ** *p* ≤ 0.01 and **** *p* ≤ 0.0001 versus Control; #### *p* ≤ 0.0001 versus ROT.

To analyze the effect of epoxidiol on the formation of free radicals in the brains of experimental animals, the lipid peroxidation level in mouse brain homogenates was studied. It was found that treatment with rotenone significantly increased the malondialdehyde content, a marker of the oxidative stress intensity in the body (Figure 11). At the same time, this indicator was 0.453 nmol/mg protein compared to 0.410 nmol/mg protein in control samples (*p* = 0.003 vs. Control). In turn, in the brain samples of animals treated with epoxidiol, there was a significant decrease in the lipid peroxidation level up to 0.342 nmol/mg protein in the case of a 21-day treatment regimen of the compound. It should be noted that in addition to significant differences compared to the ROT group (*p* < 0.0001), the epoxidiol injection significantly reduced the malondialdehyde content and when compared with the control (*p* = 0.0005 for ROT + E-diol (I) and *p* < 0.0001 for ROT + E-diol (II) vs. Control), which suggests the presence of antioxidant properties for the studied compound.

## 3. Discussion

Mitochondria, which control energy metabolism, generation of reactive oxygen species and release of apoptotic factors, play a key role in the processes of survival and apoptotic cell death. Disruption of the functioning of these organelles is an early manifestation of almost all neurodegenerative diseases [40,41], including Parkinson’s disease [42,43,44]. For more than 30 years, mitochondrial dysfunction has been considered a key factor leading to the loss of dopaminergic neurons in the substantia nigra of the brain of patients with both sporadic and familial forms of Parkinson’s disease [45,46]. This is evidenced by a large amount of experimental data, as well as the results of clinical and preclinical studies [47,48,49,50,51,52]. In this regard, a promising direction with high potential in the development of pharmacological approaches for the prevention and treatment of Parkinson’s disease is the development of therapeutic strategies aimed at maintaining the function of mitochondria [53,54].

To date, there are several experimental models used to reproduce Parkinson’s disease. These methods are based on the introduction of toxic chemical compounds, the purpose of which is to simulate the pathological conditions observed in this disease. Such substances primarily include 1-methyl-4-phenyl-1,2,3,6-tetrahydropyridine (MPTP), the use of which, however, has some limitations precisely in the context of considering mitochondrial dysfunction as the main mechanism underlying Parkinson’s disease. In this regard, in our work, another neurotoxic compound, rotenone, which is a classic inhibitor of mitochondrial complex I, was used to investigate the possibility of using the monoterpenoid epoxidiol as a potential antiparkinsonian agent. So, in the work of Zhang et al. [55], it was shown that the mitochondrial-dependent oxygen consumption and the activity of the NADH dehydrogenase enzyme in the substantia nigra were at a significantly lower level in the rotenone group compared to MPTP. This indicates that rotenone makes it possible to more accurately reproduce the pathological sign associated with mitochondrial dysfunction, without loss of effectiveness in relation to neurobehavioral reactions.

A number of studies have shown that rotenone induces cell death along the path of mitochondrial-dependent apoptosis, increasing the number of apoptotic cells [25,56,57]. In the present study, the effect of diol and its epoxidized form on the survival of the neuroblastoma cell line SH-SY5Y exposed to rotenone was studied. Neuronal-like SH-SY5Y cells are one of the most frequently used models for the study of neurotoxic and neuroprotective effects of compounds [58,59]. Our results confirmed the ability of rotenone to induce toxicity in SH-SY5Y cells. Treatment with rotenone for 24 h resulted in the death of SH-SY5Y in a dose-dependent manner, and at a toxin concentration of 400 nM, ~ 60% cell viability was observed. Such conditions simulate the situation observed in the early stages of Parkinson’s disease, when the death of about 50% of neurons in the substantia nigra is recorded, but most of them are subject to subcellular stress [60]. In our work, the preliminary 24 h incubation of epoxidiol led to a significant increase in the number of living cells in a dose-dependent manner. At the maximum studied concentration of 100 µM, this compound was able to protect SH-SY5Y cells from damage caused by rotenone, increasing viability to the values of control samples.

The toxicity shown for rotenone can be induced by apoptosis using various mechanisms, among which the key is its ability to lead to the dissipation of the mitochondrial transmembrane potential. This correlates with the pathological condition in Parkinson’s disease, when, due to a deficiency of PTEN-induced kinase 1 (PINK1), there is a decrease in the basement membrane potential and an impairment of calcium homeostasis [61], leading to the vulnerability of neurons to the opening of a transitional permeability pore, followed by the death of nerve cells [62]. When monitoring representative tracks showing the dynamic reaction of the transmembrane potential in response to the sequential addition of subthreshold nontoxic concentrations of rotenone, we detected a step-by-step increase in the fluorescence signal. This indicated a time-increasing depolarization process in response to rotenone pulses, which is consistent with the data known for this toxin [63]. On the contrary, for epoxidiol, there was a pronounced ability to retain an electric gradient on the membrane of mitochondria exposed to the toxin. Obviously, this may explain its protective role in neurotoxicity conditions on the neuroblastoma cell model due to the modulation of events in the apoptotic cascade following the loss of mitochondrial membrane potential.

As mentioned above, rotenone is a potent inhibitor of the mitochondrial electron transfer chain complex I, blocking the subsequent use of oxygen during oxidative phosphorylation and reducing the ATP production [64,65]. Such modulation of metabolism and respiratory capacity of organelles has a pronounced correlation with cell death, which is important for the formation of pathology in Parkinson’s disease [66,67,68,69]. This is due to the fact that the NADH-dehydrogenase complex is higher in the mitochondrial electron transfer chain, due to which electrons are transferred from nicotinamide adenine dinucleotide to lower molecules [70]. A number of studies show that the mitochondrial complex I dysfunction occupies a central place in the pathogenesis of Parkinson’s disease. In our work, it was found that the treatment of neuroblastoma cells with a non-toxic concentration of rotenone leads to a decrease in the oxygen consumption of the SH-SY5Y culture as a result of the function of complex I inhibition. It is not surprising that with the further addition of modulators to the system, no pronounced responses were observed in samples with rotenone, as shown in the control. This confirms the statement about the key role of blocking the NADH-dehydrogenase complex in the subsequent cascade of mitochondrial respiration events [71,72,73]. More importantly, epoxidiol significantly weakened the effect of rotenone, which indicates the ability of this compound to protect the respiratory function of organelles from the toxin action.

Thus, the results obtained as part of a phased in vitro screening using biological objects of different organization levels, demonstrated for epoxidiol the ability to inhibit the rotenone toxicity, due to alleviating mitochondrial dysfunction. Next, the effect of the compound on the parameters of mice motor function and spatial memory was investigated in a rotenone-induced model of Parkinson’s disease in vivo.

Due to the fact that the observed differences in the pharmacological effects of rotenone when using different doses require an accurate choice of the protocol for the use of the toxin, an analysis of the experimental data available to date was initially carried out. It was found that excessively high doses of rotenone lead to the development of obvious systemic toxicity affecting internal organs and causing death of the body, and do not induce the phenotype of parkinsonism [22,74]. In turn, when using low doses of the toxin with repeated administration a clear time dependence of the development of Parkinsonian pathology is observed [75,76,77], which led to the selection of the rotenone administration scheme in an in vivo experiment.

In our study, intraperitoneal administration of rotenone at a 1 mg/kg dose per day for 21 days significantly worsened the motor functions and endurance of mice. This was manifested in a decrease in motor activity in the Open Field test and motor coordination and endurance in the Accelerating Rotarod test, which, as can be assumed, mimic hypokinesia, rigidity and violation of postural reflexes observed in patients with Parkinson’s disease [78,79]. It is noteworthy that when epoxidiol was administered according to the first scheme (starting from the 8th day of the experiment, when the formation of pathology had already begun), there was a tendency to alleviate motor disorders and coordination in mice with Parkinsonism. In turn, 21-day treatment with this compound reduced motor dysfunction, which obviously implies the most pronounced success of the use of epoxidiol as a preventive therapeutic approach, as well as at the earliest stages of the disease development.

In addition to the symptoms associated with motor disorders, so-called non-motor disorders are observed in patients with parkinsonism, among which cognitive dysfunctions are the most common [80,81,82]. In particular, a large number of papers describing the pathological phenotype of Parkinson’s disease in both humans and animal models indicate an impairment of hippocampus-dependent spatial memory [83,84,85]. As part of our study in the Y-shaped maze test, mice receiving rotenone spent significantly less time in the correct maze arm, which indicates their impaired ability to learn and form memory. Interestingly, epoxidiol leveled this non-motor sign of Parkinsonism, which is an important indicator of the Parkinson’s disease progression [86].

Summarizing the results of an in vivo study of the epoxidiol effects on the neurobehavioral characteristics of mice simulating Parkinson’s disease, the combined use of the compound from the first day of rotenone administration significantly improved the behavioral status in animals, which may be due to its neuroprotective effect due to mitoprotective properties.

At the end of in vivo testing, in order to form a full understanding of the mechanisms of the antiparkinsonian action of epoxidiol, we analyzed brain samples from mice of experimental groups.

Despite the fact that various types of neuronal cells are affected in Parkinson’s disease, the role of dopamine neurons has been best studied up to date [87]. Pathophysiological studies of patients with this disease indicate that the cardinal signs, manifested primarily in the progressive development of motor symptoms, are caused by a decrease in dopamine levels and the loss of dopaminergic neurons in the nigrostriatal system [88,89]. To study the protective effect of epoxidiol on dopaminergic neurons, we performed tyrosine hydroxylase immuno-staining. The results showed that compared with the control group, mice treated with rotenone had a serious loss of TH-positive neurons. These data are completely consistent with the results of a large number of similar studies, where rotenone selectively damaged neurons in dopamine-rich brain areas. In turn, treatment with epoxidiol facilitated this situation due to the protective effect on neuronal cells in the areas most affected by Parkinson’s disease—the compact substantia nigra and the ventral tegmental area of animals with Parkinsonism. Thus, there is a direct correlation of the results obtained with the data of in vivo series of experiments, where the administration of epoxidiol significantly improved the neurobehavioral profile of mice with parkinsonism caused by rotenone.

Due to the fact that Parkinson’s disease is associated with disorders in the mitochondrial respiratory chain, which is the main source of reactive oxygen species (ROS) formation, post mortem analysis of brain samples of patients with Parkinsonism proves that dopaminergic neurons are in a condition of permanent oxidative stress and undergo radical oxidation with the ROS formation [90,91,92]. In turn, reactive oxygen species convert dopamine into reactive dopamine-quinone, which is highly toxic, and which, apparently, may be the cause of pronounced death of dopaminergic neurons [93]. A similar pattern is observed with the action of rotenone. Blocking of the complex I of the respiratory chain and, as a consequence, the transfer of electrons to oxygen leads to the formation of reactive oxygen species, especially superoxide radicals [94]. This was confirmed in our work, which shows that the administration of low rotenone doses for 3 weeks led to the disruption of the mitochondrial respiratory chain complexes and, as a consequence, an imbalance of redox homeostasis in the brain of mice, which was expressed in an increase in the level of malondialdehyde, a marker of lipid peroxidation. In turn, epoxidiol markedly reduced the generation of reactive oxygen species induced by rotenone, which suggests the presence of a protective antiparkinsonian mechanism of epoxidiol associated with the ability to reduce oxidative stress.

## 4. Materials and Methods

### 4.1. Agents

Diol and E-diol were synthesized from (-)-verbenone (Sigma-Aldrich, St. Louis, MO, USA) according to earlier published methods [17,18] with the purity > 98%.

### 4.2. Preparation of Working Solutions

To obtain initial solutions of the studied compounds in a 10 mM working concentration, diol and epoxidiol were dissolved in sterile bidistilled water. Rotenone solution (400 µM, Sigma-Aldrich, St. Louis, MO, USA) it was prepared in dimethyl sulfoxide (DMSO, Sigma-Aldrich, St. Louis, MO, USA). The obtained solutions were stored at a temperature of +2 to +4 °C. Methods of processing cells or organelles with compounds are indicated in the relevant subsections of this section and the captions to the figures.

### 4.3. Cell Lines and Cultivation

The human neuroblastoma cell line SH-SY5Y provided by the Institute of Cytology of the Russian Academy of Sciences was cultured in a humidified atmosphere with 5% CO_2_ at +37 °C. Cells were grown in Dulbecco’s Modified Eagle Medium (DMEM) (Gibco, Scotland, UK) containing 10% fetal bovine serum (ThermoFisher Scientific, Paisley, UK), L-glutamine (2 mM) (Gibco, Scotland, UK), and penicillin-streptomycin (1% by volume) (PanEco, Moscow, Russia). The nutrient medium was changed every 2–3 days after reaching 85–90% of cell growth.

### 4.4. Cell Viability Assay

The human neuroblastoma cell line SH-SY5Y (provided by the Institute of Cytology of the Russian Academy of Sciences) was seeded into 96-well plates (1 × 10^4^ cells/200 µL, Corning Inc., New York, NY, USA) in a growth medium (composition described above) and cultured for 24 h at +37 °C, 5% CO_2_. A day later, diol and epoxidiol were added (1, 10, 50 and 1.0 and 100 µM). Rotenone (100 nM and 400 nM) was introduced into the wells of the plate 24 h after the addition of the studied compounds and incubated for another 24 h. The negative control was treated with appropriate volumes of solvents used to obtain working solutions of diol and epoxidiol (bidistilled H_2_O) and rotenone (DMSO), and the positive control was bidistilled H_2_O and rotenone. Cell viability was assessed by MTT analysis, as described in [95]. To do this, MTT (bromide 3-(4,5-dimethylthiazole-2-yl)-2,5-diphenyltetrazolium, 5 mg/mL, Dia-m, Moscow, Russia) was introduced into each well and additionally incubated for 2 h (until a characteristic color appears). Using a flatbed analyzer (Cytation3, Biotech Instruments Inc., Winooski, VT, USA), the optical density of the formed formazan granules was determined at λ = 530 nm.

### 4.5. Mitochondrial Membrane Potential Measurements

The mitochondrial transmembrane potential was measured by recording the fluorescence of the potential-dependent marker Safranin O [96] in a 96-well plate. The well of the tablet contained mitochondria (0.5 mg/mL) previously diluted in a buffer (225 mM mannitol (Dia-m, Moscow, Russia), 75 mM sucrose (Dia-m, Moscow, Russia), 10 mM HEPES (Dia-m, Moscow, Russia), 1 mM KH_2_PO_4_, 20 µM EGTA (Cabiochem, San Diego, CA, USA), pH = 7.4) mixed with 5 µM safranin O. According to the experimental scheme, the studied substances were added to the mitochondria in a concentration of 10 up to 100 µM and incubated for 5 min. After the incubation time, the initial measurement was carried out for 10 cycles. After that, substrates of the mitochondrial respiratory chain complex I were added to each well—a solution of sodium glutamate and sodium malate (5 mM). The measurement continued for the next 30 cycles until a stable signal indicating the polarization of the mitochondrial membrane appeared. Next, the rotenone was titrated with pulses in all wells (three times in 10 nM) with 20 measurement cycles after each addition. Finally, 25 µM of Ca(II) was added to achieve maximum fluorescence due to the opening of the mitochondrial pore and measured for 20 cycles. Fluorescence measurements were carried out on a Victor 3 plate analyzer (Perkin Elmer, Rodgau, Germany) at λ_ex_ = 485 nm and emission λ_em_ = 590 nm.

### 4.6. Automatic Measurement of Energy Metabolism in Real Time by Registering Oxygen Consumption Rate

The oxygen consumption rate (OCR) of the SH-SY5Y neuroblastoma cell line was measured in real time using the Seahorse XFe96 cellular metabolism analyzer (Agilent Technologies, Santa Clara, CA, USA) and the Seahorse/Agilent Mito Stress Test [38] with some modifications.

SH-SY5Y cells in the exponential growth phase were seeded into a 96-well Seahorse cell culture microplate. The planting density of the cell culture was 3 × 10^4^/well. After 24 h, solutions of diol and epoxidiol (100 µM) were added to the cells, and an equivalent volume of solvent was added to the remaining wells and left to incubate at +37 °C, 5% CO_2_ per day. The next day, according to the protocol, the sensor cartridge with injection ports was filled with reagents to assess changes in OCR by modulating cellular metabolism. Next, the analyzer was calibrated, after which the plate was replaced with a research plate with cells and the oxygen consumption rate was recorded.

Initially, three measurements of the basic OCR rate of the SH-SY5Y cell line were carried out. Then, from port A, rotenone was injected into the medium at a non-toxic concentration of 10 nM or a medium containing the appropriate solvent (as a control) and the oxygen consumption rate was measured for five cycles. Further, oligomycin (2 µM, Sigma-Aldrich, St. Louis, MO, USA), FCCP (carbonyl cyanide-4 (trifluoromethoxy) phenylhydrazone, 2 µM, Sigma-Aldrich, St. Louis, MO, USA) and antimycin A (1 µM, Sigma-Aldrich, St. Louis, MO, USA) were sequentially injected through ports B, C and D and three measurements were carried out.

The use of such a protocol made it possible to evaluate a number of parameters, including acute reaction, basal respiration, respiration after blocking the I complex of the respiratory chain of electron transfer by rotenone, respiration associated with ATP production, proton leakage, maximum respiration, etc. The acute reaction was calculated as the last OCR measurement before injection of rotenone minus the last measurement after injection into port A. The addition of oligomycin, which blocks ATP synthase and leads to the suppression of oxidative phosphorylation, made it possible to calculate the OCR associated with ATP production by subtracting the last measurement before injection of oligomycin and measuring the minimum rate after injection of oligomycin. Cells treatment of FCCP that dissociate electron transport and ATP synthesis made it possible to evaluate the maximum OCR associated with respiration and spare respiratory capacity. Antimycin A, which is an inhibitor of cellular respiration by blocking the III complex of the respiratory chain, allowed us to calculate OCR that is not associated with mitochondrial respiration.

The results were analyzed using Wave Desktop software version 2.6 (Agilent Technologies, Santa Clara, CA, USA).

### 4.7. Experimental Animals

Forty adult male mice of the C57BL/6J line (The Jackson Laboratory, Bar Harbor, ME, USA), crossing in an animal facility for several years on identical genetic background (age 14 weeks; weight 24 ± 2 g), were used for the study. The animals were kept in conditions with controlled temperature (23 ± 1 °C), humidity (50 ± 5%) and lighting cycle (12 h/12 h light/dark). Water and food were given ad libitum. All behavioral tests were conducted in the test room at the same time of day. On the day of testing, one hour before the start of the experiment, the mice were moved to the experimental room, and the animals were allowed to adapt to the environment.

By simple aleatorization, four groups of mice were formed (*n* = 10 per group):

(1) Control—a group of mice that were intraperitoneally injected with bidistilled water and a solution of NaCl + 10% DMSO—1 µL/g/day (for 21 days);
(2) ROT—a group of mice receiving intraperitoneal injections of bidistilled water and rotenone (1 mg/kg)—1 µL/g/day (for 21 days);
(3) ROT + E-diol (I)—a group of mice treated intraperitoneally with epoxidiol (15 mg/kg) and NaCl + 10% DMSO solution—1 µL/g/day (for 14 days (starting from the 8th day of the experiment);
(4) ROT + E-diol (II)—a group of mice treated intraperitoneally with epoxidiol (15 mg/kg) and NaCl + 10% DMSO solution—1 µL/g/day (for 21 days (starting from day 1 of the experiment).

Epoxidiol was dissolved in sterile bidistilled water immediately before the experiment. The rotenone solution was prepared in DMSO and additionally dissolved in NaCl (up to 10% DMSO).

### 4.8. Analysis of the Motor Activity of Mice in the Open Field Test

An Open Field test was used to assess the motor activity of mice. The installation was a square gray box with a floor size of 40 × 40 cm and walls 40 cm high (the lighting intensity was 50 lux). The animal was placed in the center of the arena to study the installation for 5 min, after which the mouse was returned to the holding cage. After each test, the open-field arena was cleaned with 70% ethanol in order to remove any odors from the previous animal and thoroughly dried. Each test was recorded using a camera connected to a computer, which subsequently allowed the results to be processed using the EthoVision XT system software (Noldus, Wageningen, The Netherlands). The parameters of motor function were evaluated, such as average speed and distance traveled.

### 4.9. Assessment of the Motor Function and Endurance of Animals in the Accelerating Speed Rotarod Test

The motor function of animals was assessed on a rolling rod in the accelerating speed Rotarod test. The Rotarod hardware and software complex (Ugo Basile 7650, Biological Research Apparatus, Italy) is a cylinder divided by circular partitions into individual compartments and rotates cylindrical rods (3 cm in diameter) at a given speed. The installation makes it possible to assess motor and coordination disorders by the ability of animals to stay on a rotating cylinder.

For training, on the first day of the experiment, mice were placed on a rolling rod for 5 min at a constant speed (4 rpm), after which they were returned to the holding cell. After 24 h, each animal was tested four times in accelerating mode (from 4 to 40 rpm) with 30 min intervals between trials, giving the mouse a maximum of 5 min to try. Latency to fall in each trial was recorded. The average latency to fall value for all four trials was included in the final statistics. After each animal, the apparatus was cleaned with 70% ethanol and air-dried.

### 4.10. Analysis of Hippocampus-Dependent Spatial Memory of Mice in the Y-Maze Test

The analysis of hippocampus-dependent spatial memory of animals was carried out in the Y-maze test. The installation of the Y-maze test is made of acrylic with three arms (arm size 32.5 × 8.5 × 15 cm) located at a distance of 120° from each other. During the training phase, the mouse was placed at the beginning of one maze arm (the starting arm) and allowed to move freely through two open arms of the maze for 5 min. After 30 min, a testing phase was carried out, during which the animal was placed in the same starting position but allowed to examine all three arms of the installation. After each animal, all the arms of the maze were thoroughly cleaned with 70% ethanol and dried. All attempts were recorded on video for subsequent processing using the EthoVision XT system (Noldus, Wageningen, the Netherlands). The duration of the stay of the animals during the testing phase in the maze arms was analyzed. As a criterion for the effectiveness of the spatial memory formation, the animals’ presence in a new arm of the maze was considered.

### 4.11. Preparation of Histological Sections, Immunohistochemistry and Counting of Neuronal Cells

Four-month-old male mice were terminated, and their brains were dissected. Fixation, preparation of histological sections, staining with anti-tyrosine hydroxylase antibody (TH, mouse monoclonal antibody, clone TH-2, Sigma diluted 1:1000) and stereological counting of TH-positive neurons in the SNpc and ventral tegmental area (VTA) were performed as described [97,98].

Briefly, the margins of SNpc and VTA on stained sections were outlined using distribution atlas of TH-positive cells [99]. The first section for counting was randomly chosen from the first ten sections that included the SN/VTA region. Starting from this section, on every fifth section, TH-positive cells with a clearly visible nucleus were counted through the whole region. ZEN Microscopy Software (Carl Zeiss) was employed to measure diameters of 30 nuclei of dopaminergic neurons in each of these regions of every mouse brain included in this study. The nuclei were chosen randomly, and the distance measured as the horizontal length as they appeared on the screen. A mean was calculated for each animal and used for Abercrombie’s correction [100] to obtain an actual number of TH positive cells in the structure.

### 4.12. Evaluation of Bioenergetic Parameters of the Mitochondrial p2 Fraction

The study of the electron transport chain complexes was carried out on a preparation of the brain mitochondrial p2 fraction obtained by differential centrifugation. The Agilent Seahorse XF96e analyzer (Seahorse Bioscience, North Billerica, MA, USA) was used to measure the rate of oxygen uptake by organelles under the action of modulators. A total of 10 micrograms of mitochondria were loaded into the well of the tablet and a cold analysis buffer was added (1xMAS: 220 mM D-mannitol, 70 mM sucrose, 10 mM KH_2_PO_4_, 5 mM MgCl_2_, 2 mM HEPES, 1 mM EGTA, 0.2% bovine serum albumin, free of fatty acids, pH = 7.2). The tablet was centrifuged at 2000× *g* for 20 min at 4 °C. Then, a warm 1xMAS buffer containing 10 mM of sodium malate and 10 mM of sodium glutamate was added to each well. Mitochondrial electron flow was evaluated by sequentially adding an inhibitor of the complex I—rotenone (2 µM), a substrate of the comp—lex II—sodium succinate (2 µM), an inhibitor of the complex III—antimycin A (1 µM) and substrates of the complex IV—ascorbate/N,N,N’,N’-tetramethyl-p-phenylenediamine dihydrochloride (TMPD) (0.5 µM).

### 4.13. Study of the Intensity of Lipid Peroxidation in Mouse Brain Homogenates

The study of the intensity of lipid peroxidation (LPO) was carried out using a modified version of the TBA test. This technique is based on the reaction of 2-thiobarbituric acid with intermediate LPO products, as a result of which a colored trimethine complex is formed, the main role in the formation of which belongs to malonic dialdehyde.

The intensity of LPO was determined in mouse brain homogenates. To do this, mice were sacrificed using the method of cervical dislocation and the brain was extracted, half of which was homogenized in a buffer containing 120 mM KCl, 20 mM HEPES pH = 7.4, at 4 °C, centrifuged at 1500 rpm and the supernatant was selected. The resulting homogenate (4 mg/mL) was introduced into the wells of a deep-well plate and a reagent for TBA-reactive products was added to each sample, after which it was incubated for 90 min at 90 °C. After the incubation time, the samples were centrifuged at 6000 rpm for 20 min and the optical density of the selected supernatant was measured on a flatbed analyzer (Cytation3, Biotech Instruments Inc., Winooski, VT, USA) λ = 540 nm.

## 5. Conclusions

Parkinson’s disease is a multifactorial disease and is characterized by heterogeneous symptoms, including classical motor disorders and non-motor features caused by the loss of dopaminergic neurons in the brain substantia nigra. One of the key roles in the pathogenesis of this disease belongs to disorders in the functioning of the NADH-dehydrogenase complex, which is a trigger in starting the process of oxidative phosphorylation and ATP production by mitochondria. And due to the fact that currently there are no treatment methods that would slow down or stop the neurodegenerative process in Parkinson’s disease, the urgent task of modern biomedicine is to search for new drugs, in particular, due to the ability to modulate mitochondrial dysfunction. In this study, the analysis of the antiparkinsonian properties of trans-epoxide (1S,2S,3R,4S,6R)-1-methyl-4-(prop-1-en-2-yl)-7-oxabicyclo [4.1.0]heptane-2,3-diol (epoxidiol) on a model of rotenone-induced neurotoxicity using in vitro, in vivo and ex vivo approaches in the context of studying the mitoprotective properties of the compound. Our results showed that epoxidiol had cytoprotective properties on the SH-SY5Y cell line exposed to rotenone. This may be due to the ability of the compound to prevent the loss of mitochondrial membrane potential and, as a consequence, to modulate events in the subsequent apoptotic cascade. The analysis of the bioenergetic profile of neuroblastoma cells showed for epoxidiol the ability to restore the rate of oxygen consumption after inhibiting the complex I function and, as a consequence, weaken the effect of rotenone. In the conditions of modeling Parkinson’s disease in vivo, treatment with epoxidiol led to the leveling of both motor disorders and a non-motor symptom—cognitive dysfunction. In conclusion, the post mortem analysis of animal brain samples demonstrated the ability of epoxidiol to prevent the loss of dopaminergic neurons, which may be due to its properties to restore the functioning of mitochondrial respiratory chain complexes and significantly reduce the production of reactive oxygen species. Thus, the results obtained indicate that epoxidiol can be considered as a new agent for the treatment of Parkinson’s disease and allow us to hope for their further translation into the practical plane of the development of promising pharmacological substances for the treatment of this disease.

## Data Availability

Samples of the compounds and data used during the current study are available from the corresponding author.
